# Selection and Validation of qRT-PCR Reference Genes in *Elsholtzia stauntonii*

**DOI:** 10.3390/cimb48090933

**Published:** 2026-09-12

**Authors:** Miaomiao Liu, Xiangyu Lin, Xiumei Zhou

**Affiliations:** Henan Engineering Research Center of the Development and Utilization of Characteristic Horticultural Plant, School of Horticulture and Landscape Architecture, Henan Institute of Science and Technology, Xinxiang 453003, China; linyu7252@163.com (X.L.); zxm@hist.edu.cn (X.Z.)

**Keywords:** *Elsholtzia stauntonii*, reference gene stability, gene expression normalization, geNorm, NormFinder, Comparative ΔCt, BestKeeper, RefFinder

## Abstract

Selecting and validating appropriate reference genes is a prerequisite for accurate gene expression analysis by means of quantitative real-time PCR (qRT-PCR), particularly in plant species with limited molecular research. *Elsholtzia stauntonii* is an economically important ornamental, medicinal, and aromatic plant. Although it exhibits a good tolerance to cold and drought, it is notably susceptible to waterlogging stress. To date, systematic investigations into its gene expression in different tissues and under waterlogging stress have not been extensively performed. In the present study, eight candidate reference genes, including *GAPDH*, *ACT*, *UBQ*, *UBC*, *TUA*, *TUB*, *EF1-α*, and *eIF3K*, were investigated using qRT-PCR in 13 samples from two distinct groups. The expression stability of these candidate genes was comprehensively assessed using the most commonly adopted algorithms: geNorm, NormFinder, Comparative ΔCt, BestKeeper, and RefFinder. The results demonstrated that *UBC* and *EF1-α* were the most suitable reference genes for different tissue samples, whereas *ACT*, *GAPDH*, and *UBQ* exhibited optimal reference genes for leaves under waterlogging stress. In contrast, *eIF3K* and *TUB* were identified as the least stable reference genes in different tissue samples and waterlogged leaves, respectively. To validate the suitability of the selected reference genes, the expression patterns of phenylalanine ammonia-lyase gene 1 (*ElstPAL1*) were analyzed. Comprehensive consideration of reference gene performance and the validation results confirmed that *GAPDH* and *ACT* were applicable and accurate for qRT-PCR normalization for different tissues of *E. stauntonii*, while *ACT*, *GAPDH*, and *UBQ* were effective for waterlogging-stressed leaves. These findings establish a solid foundation for future gene expression studies in *E. stauntonii* samples from different tissues and subjected to waterlogging stress and will facilitate future molecular studies in this economically important species.

## 1. Introduction

Owing to its high specificity, rapidity, and sensitivity, quantitative real-time PCR (qRT-PCR) has emerged as a powerful tool for quantifying the transcript abundance of target genes in various organisms [1]. However, the accuracy and reliability of qRT-PCR analyses are greatly affected by RNA quality, template quantity, and enzyme reaction efficiency, highlighting the importance of selecting a reliable reference gene for qRT-PCR data normalization [2]. Over the last few decades, a series of reference genes, such as glyceraldehyde-3-phosphate dehydrogenase (*GAPDH*), actin (*ACT*), and ubiquitin (*UBQ*), have been extensively utilized to normalize gene expression data in plants [3,4,5,6,7]. An ideal reference gene should, in theory, maintain expression stability under varying conditions and remain unaffected by any experimental treatment [8,9]. Regrettably, in many cases, the expression levels of reference genes fluctuate greatly among different species or under various conditions [10]. For example, *UBQ* was established as the optimal reference gene during floral induction in sugarcane, while *GAPDH* was the least stable [11]. Similarly, *ACT* was recommended as the reference gene under PEG treatment in *Glehnia littoralis*, while *GAPDH* and *UBQ* proved unsuitable for data normalization [12]. To date, no universal reference gene has been identified across all plant species and experimental settings [1]. To address this gap, reference genes with relatively stable expression should be systematically screened and validated for each specific organism and experimental condition.

*Elsholtzia stauntonii* Benth. (mint bush), a perennial sub-shrub belonging to the *Lamiaceae* family, is widely distributed in China [13]. Its aerial parts are highly aromatic and rich in essential oils, which exhibit significant insecticidal activity against certain pests and have been developed as novel biological insecticides [14,15,16,17]. The branches and leaves of *E. stauntonii* are traditional Chinese medicinal materials utilized for the treatment of the common colds, toothaches, dysentery, and digestive system diseases [13,18,19,20]. The results of phytochemical investigations have revealed that this species is abundant in various secondary metabolites, such as muxiangrine I, muxiangrine II, muxiangrine III, cuminol, caryophyllene, and 1,8-cineole [13,16,17,21]. The results of recent studies revealed that its essential oils can inhibit pathogens responsible for bovine mastitis, positioning them as a promising phytotherapeutic alternative [22]. Moreover, *E. stauntonii* is suitable for urban greening and serves as a wild nectar plant due to its dense inflorescences and vibrant floral colors [23]. In 2019, it was designated as one of the main greening shrub species in Beijing. Although *E. stauntonii* exhibits strong tolerance to drought and barren environments [24], it is notably susceptible to waterlogging stress, which poses a major threat to its survival. Consequently, elucidating the molecular mechanism underlying the responses of *E. stauntonii* to waterlogging stress is crucial and would facilitate the molecular breeding of waterlogging-tolerant cultivars.

To date, reference gene validation has been investigated and reported in at least 12 *Lamiaceae* species from genera including *Salvia* [25], *Lamiophlomis* [26], *Scutellaria* [27], and *Isodon* [28]; however, no study has focused on *Elsholtzia*. The published literature consistently demonstrates the necessity of reference gene validation. As a species of *Lamiaceae*, the whole genome of *E. stauntonii* has yet to be sequenced. The lack of genomic information has severely impeded the identification and validation of suitable reference genes for this species. This limitation can be effectively addressed by RNA sequencing (RNA-seq). RNA-seq can rapidly acquire all of the transcript information of a sample at a specific temporal point under a given condition [29]. It has been proven to be an efficient method for mining and screening genetic resources, making it particularly suitable for non-model plant species lacking a reference genome [30,31]. To date, RNA-seq has been extensively used in the systematic screening of reference genes in various plant species [32,33]. In this work, eight candidate reference genes, including *GAPDH* (glyceraldehyde-3-phosphate dehydrogenase), *ACT* (actin), *UBQ* (ubiquitin), *UBC* (ubiquitin-conjugating enzyme), *TUA* (α-tubulin), *TUB* (β-tubulin), *EF1-α* (elongation factor 1), and *eIF3K* (eukaryotic translation initiation factor 3 subunit K), were initially selected from the transcriptome of *E. stauntonii*. We hypothesized that these candidate genes would exhibit varying expression stability across different tissues or stress conditions rather than being constitutively stable in *E. stauntonii*. To test this hypothesis, the expression stability of eight candidates was systematically evaluated in two distinct sample groups (different tissues and waterlogging groups). Ultimately, the expression patterns of *ElstPAL1*, the first committed enzyme in the phenylpropanoid pathway [6], were verified in these samples to further validate the reliability of the selected reference genes. These findings will provide a solid foundation for further explorations into key functional genes in response to waterlogging stress and facilitate future molecular work in this economically important species.

## 2. Materials and Methods

### 2.1. Plant Materials, Treatments and Samples

The *E. stauntonii* plants used in this study were taxonomically authenticated by Associate Research Fellow Bing Li (Institute of Botany, Chinese Academy of Sciences). A voucher specimen of *E. stauntonii* has been deposited at the China National Botanical Garden (ID cvbg01141026). All experimental procedures were performed in accordance with local and national regulations. The plant materials were cultivated in the field nursery of Henan Institute of Science and Technology (35.18° N, 113.55° E), Xinxiang, Henan province, China. The expression stability of the candidate reference genes was assessed in two distinct sample sets: (1) different tissues (Dt) containing roots, stems, leaves, flowers, and embryogenic calli; (2) leaves under waterlogging stress (Ws). The different tissues were sampled from annual shoots. Embryogenic calli were induced from mature leaves following the protocol described below. Firstly, robust mature leaves were excised from median nodes of the branches. After thorough rinsing under running water, the leaves were surface-sterilized with 0.1% HgCl_2_ for 5–8 min, followed by 5–6 washes with sterile distilled water. Subsequently, the sterilized explants were aseptically sectioned into 0.5 cm^2^ disks and cultured on Murashige and Skoog (MS) basal medium supplemented with 1.0 mg/L 6-benzylaminopurine (6-BA) and 1.0 mg/L α-Naphthaleneacetic acid (NAA) (Solarbio, Beijing, China). The cultures were maintained in a tissue culture room under a photoperiod of 16 h light/8 h dark at 20–22 °C, with a light intensity of 8000 lx and relative humidity of 65–75%.

Waterlogging stress experiments were conducted under greenhouse conditions. Rooted cuttings were initially cultured for three weeks in a soil mixture comprising Denmark Pindstrup substrate and China-VMC vermiculite (3:1, *v*/*v*). After rooting, they were transplanted into 15 cm diameter plastic pots containing fresh substrate and grown under normal cultivation conditions for an additional month. Plantlets were watered every 4 days to maintain the relative soil humidity at 65–75% and fertilized biweekly. Thereafter, uniform-sized and robust plantlets were selected and randomly divided into control and waterlogging stress groups. For the waterlogging treatment, pots were placed in a container filled with tap water maintained at 3 cm above the soil surface. The water level was sustained by daily replenishment, creating a waterlogging stress condition. The control plants were irrigated normally to maintain optimal soil moisture without drought or waterlogging stress. The treatments lasted 5 days; fresh leaves from both the control and stress groups were sampled at 0 h, 6 h, 12 h, 1 d, 2 d, 3 d, 4 d, and 5 d. All samples were immediately stored in liquid nitrogen after collection. Each treatment comprised three biological replicates, with technical triplicates per biological replicate.

### 2.2. Total RNA Isolation and Reverse Transcription

Total RNA was extracted using a FastPlant Total RNA Extraction Kit (Nobelab, Beijing, China). RNA integrity was assessed by means of 1.5% agarose gel electrophoresis, with distinct 28S and 18S rRNA bands confirming minimal degradation. To eliminate potential genomic DNA contamination, all RNA samples were treated with RNase-free DNase I (TaKaRa, Otsu, Shiga, Japan). Subsequently, they were quantified precisely using a microspectrophotometer (NanoDrop-2000c, Thermo Fisher, Waltham, MA, USA), and the OD260/OD280 ratio was measured to verify their purity. First-strand cDNA was synthesized from 1000 ng of high-quality total RNA using the PrimeScript RT reagent Kit (TaKaRa, Otsu, Shiga, Japan) with 50 ng of random hexamer primers. The resulting cDNA products were immediately stored at −80 °C until further use.

### 2.3. Candidate Reference Gene Selection and Primer Design

Eight candidate reference genes, including *GAPDH*, *ACT*, *UBQ*, *UBC*, *EF-1α*, *TUA*, *TUB*, and *eIF3K*, were selected based on their FPKM (>10) and fold change values (<2) from the transcriptome data of *E. stauntonii* plants (PRJNA1241349) [34]. Gene-specific primers for qRT-PCR were designed using NCBI primer-BLAST (http://www.ncbi.nlm.nih.gov/tools/primer-blast/ accessed on 27 June 2025) according to the following criteria: primer length, 18–23 bp; GC content, 40–60%; melting temperature (Tm), 58–62 °C; amplicon length, 90–200 bp [35].

### 2.4. qRT-PCR and Primer Specificity Testing

qRT-PCR assays were conducted on a Bio-Rad CFX Connect Real-Time PCR Detection System (Bio-Rad, Hercules, CA, USA) using the RealStar Fast SYBR qPCR Mix (GenStar, Beijing, China). The original cDNA was diluted to a final concentration of 100 ng/μL and used as the template. According to the instructions, each 20 μL reaction mixture contained 100 ng of cDNA, 10 μL of 2× SYBR qPCR mix, 1 μL each of forward and reverse gene-specific primers (10 μmol/L), and the right amount of nuclease-free ddH_2_O. The amplification program was as follows: 95 °C for 30 s, 39 cycles of 95 °C for 10 s, and 60 °C for 10 s, followed by 72 °C for 20 s. All qRT-PCR assays were performed according to the MIQE guidelines, and both no-template and no-reverse-transcription controls were included. To verify the specificity of each primer, conventional PCR amplification and melt curve analysis were performed. The PCR amplification efficiency (E) was calculated based on a standard curve generated using a five-fold dilution series of pooled cDNA as the template using the linear regression model. They were derived from the slope of the regression line according to the equation E = 10^(−1/slope)^ − 1 [36,37]. All qRT-PCR reactions were performed with three biological replicates and three technical replicates.

### 2.5. Stability Analysis and Validation of Candidate Reference Genes

The threshold cycle (Ct) values obtained using CFX equipment software were utilized to assess gene expression levels. Before stability analysis, raw Ct data were assessed for outliers according to the consistency of replicates. Thereafter, four widely adopted statistical algorithms, namely, geNorm [38], NormFinder [39], Comparative ΔCt [40], and BestKeeper [41], and the comprehensive stability ranking program RefFinder [42] (https://blooge.cn/RefFinder/?type=reference accessed on 8 October 2025) were used to evaluate the expression stability of candidate reference genes.

To further confirm the reliability of the selected reference genes, the expression patterns of *ElstPAL1* were analyzed using the top two or three reference genes for data normalization. Relative expression levels were calculated using the Pfaffl method, as previously described by Bustin et al. [36]. Statistical analysis was conducted using SPSS software (version 29). One-way analysis of variance (ANOVA) was performed to evaluate differences. Significant differences were defined as *p* < 0.05 (*) and *p* < 0.01 (**). Data are presented as the mean ± standard deviation of three biological replicates.

## 3. Results

### 3.1. Selection of Candidate Reference Genes and RNA Quality Assessment

Following established protocols, housekeeping genes with stable and constitutive expression were selected as reference genes. Based on RNA-seq data, eight genes (*GAPDH*, *ACT*, *UBQ*, *UBC*, *EF1-α*, *TUA*, *TUB*, and *eIF3K*) were selected as candidate reference genes. The integrity of all RNA samples was verified via agarose gel electrophoresis, which showed clear and bright 28S and 18S bands without smearing or degradation, signifying high RNA integrity (Figure S1a). The NanoDrop spectrophotometer results demonstrated that the OD_260/280_ absorbance ratios of all RNA samples ranged from 1.9 to 2.1, indicating that the RNA purity met the experimental requirements.

### 3.2. Primer Specificity Verification and Amplification Efficiency Analysis

**Figure 1:**
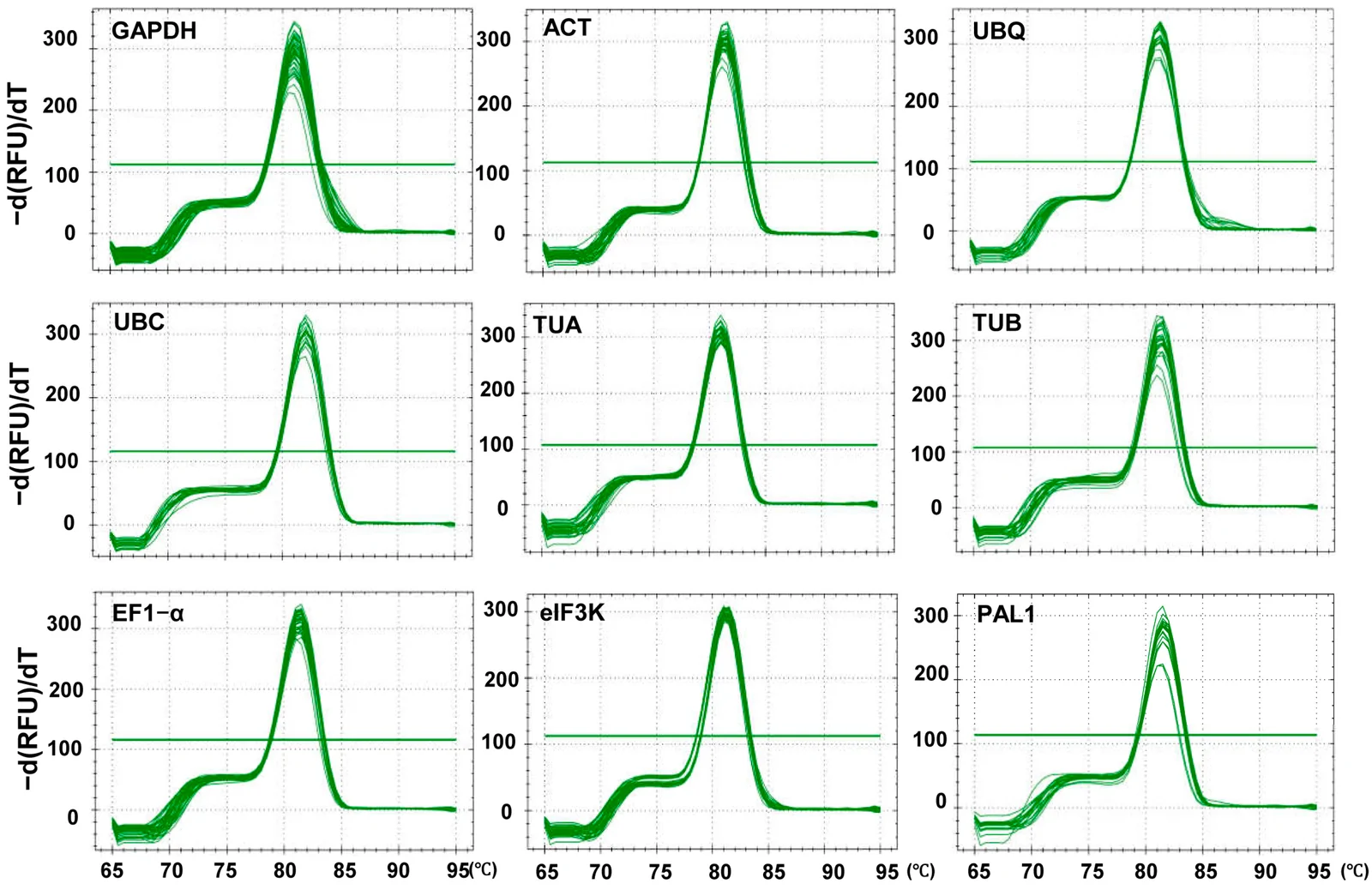
qRT-PCR amplification of eight candidate reference genes and the target gene *PAL1*. Melting curves of eight candidate reference genes and the target gene *PAL1* exhibited only single peaks. All melting curves represented data from three replicates across all samples. The green straight line represents the peak-detection threshold; only peaks exceeding this threshold were scored as valid melting peaks.

Primer specificity was tested by means of conventional PCR and qRT-PCR analysis. The amplicon sizes ranged from 100 to 150 bp (Figure S1b), consistent with the expected product lengths (Table 1). Each primer pair displayed high amplification specificity, as only a single clear band was observed in each lane (Figure S1b). Furthermore, the melting curves exhibited a single sharp specific peak, further confirming primer specificity (Figure 1). The amplification efficiency of the eight candidate reference genes ranged from 94.4% to 107.8%, and all of the coefficient of determination (R^2^) values of the standard curves exceeded 0.99 (Table 1). These results indicated that the primer pairs were reliable and suitable for subsequent qRT-PCT analysis.

### 3.3. Expression Patterns of Candidate Reference Genes

**Figure 2:**
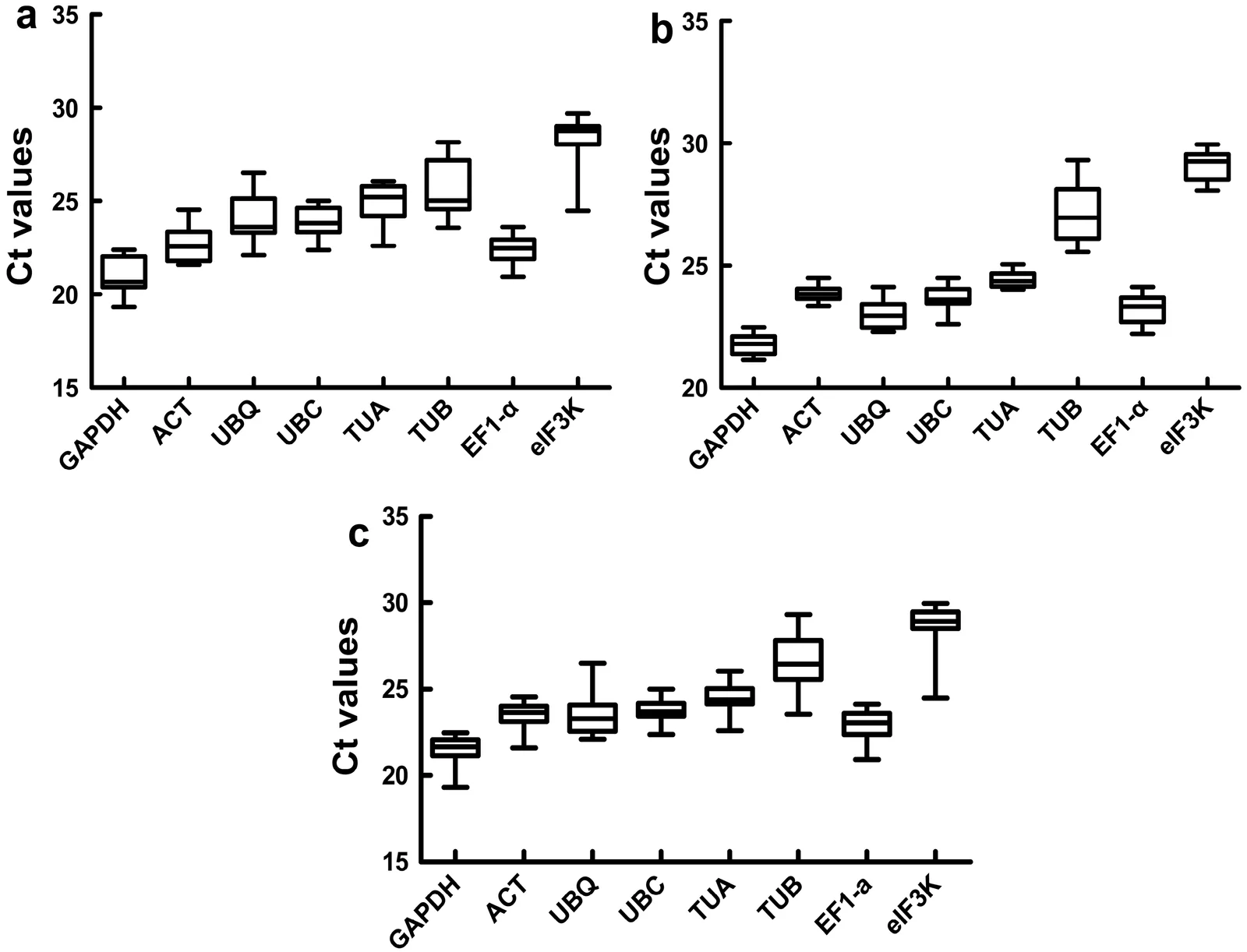
Expression levels of eight candidate reference genes in Dt, Ws and total samples. The box-plot graph exhibits the Ct values of each gene in Dt (**a**), Ws (**b**) and total (**c**) samples. The line across the box represents the median. Whiskers represent the minimum and maximum Ct values. Dt, different tissues; Ws, waterlogging stress.

A box-plot graph was generated based on Ct values (Figure 2). The mean Ct values of the eight candidate reference genes varied from 19 to 30 across Dt and Ws samples, indicating their moderate transcript abundance. Among these genes, *GAPDH* exhibited the highest expression level, with the lowest mean Ct values of 20.89 in Dt samples and 21.76 in Ws samples. By contrast, *eIF3K* exhibited the highest mean Ct values of 28.18 in Dt samples and 29.10 in Ws samples, representing the least abundant transcript (Figure 2). Based on the dispersion of Ct value distributions, *EF1-α* and *ACT* were identified as the most stable reference genes in Dt and Ws samples, respectively. *TUB* exhibited the greatest expression variation in both Dt and Ws samples. For the total samples, *UBC* was the most stable reference gene and *TUB* was the least stable. Thus, it can be concluded that the dispersion of reference genes varies across different samples. Similar results were reported in *Macadamia integrifolia* [43]. It should be noted that the Ct distribution visualized using box plots is only descriptive, and the formal stability evaluation of candidate reference genes was performed using GeNorm, NormFinder, Comparative ΔCt, BestKeeper, and RefFinder.

### 3.4. Expression Stability of Candidate Reference Genes

#### 3.4.1. geNorm Analysis

**Figure 3:**
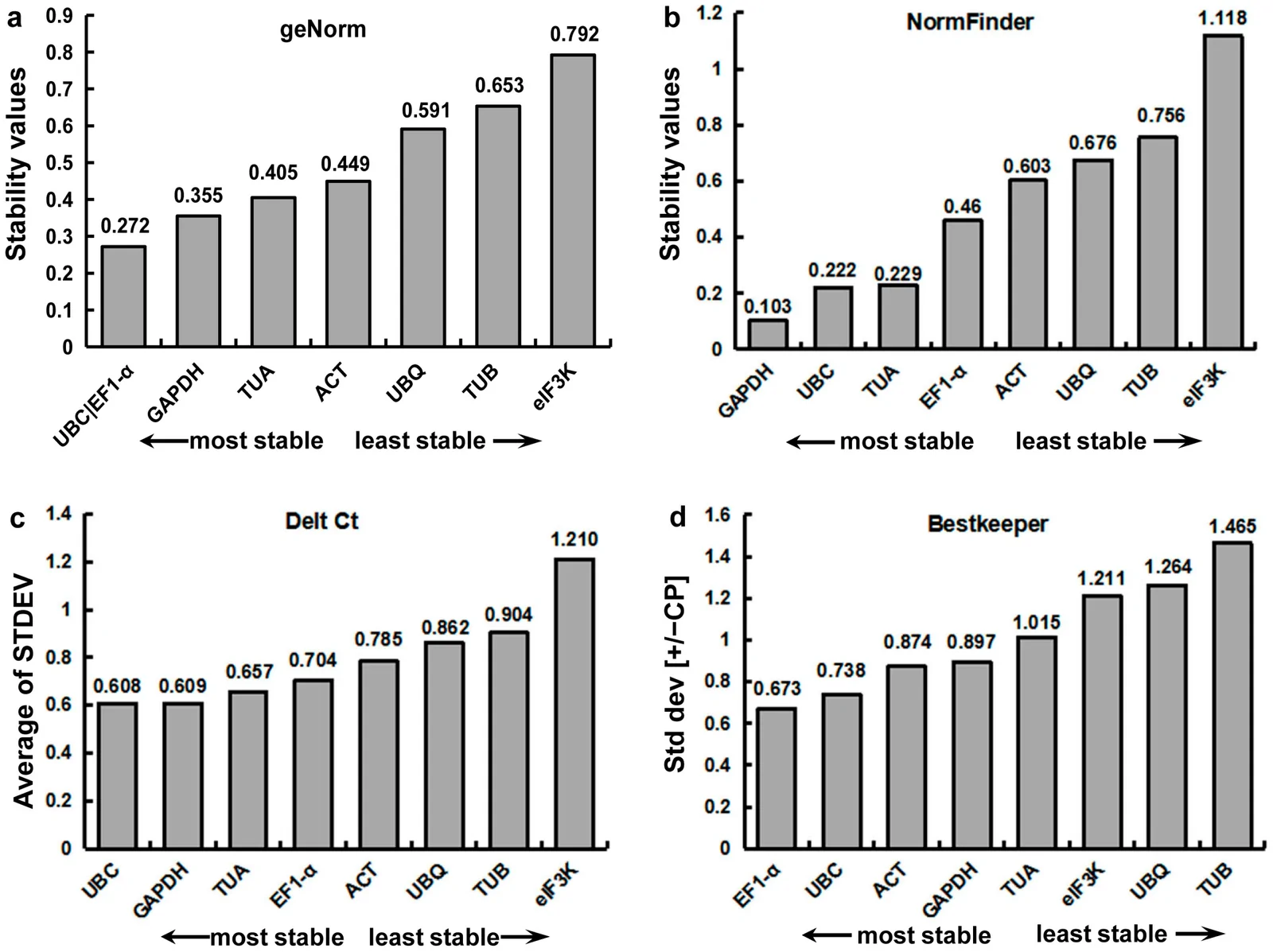
Expression stability analysis of eight candidate reference genes in Dt samples. Rank order was determined by geNorm (**a**), NormFinder (**b**), Comparative ΔCt (**c**) and BestKeeper (**d**). Dt, different tissues.

**Figure 4:**
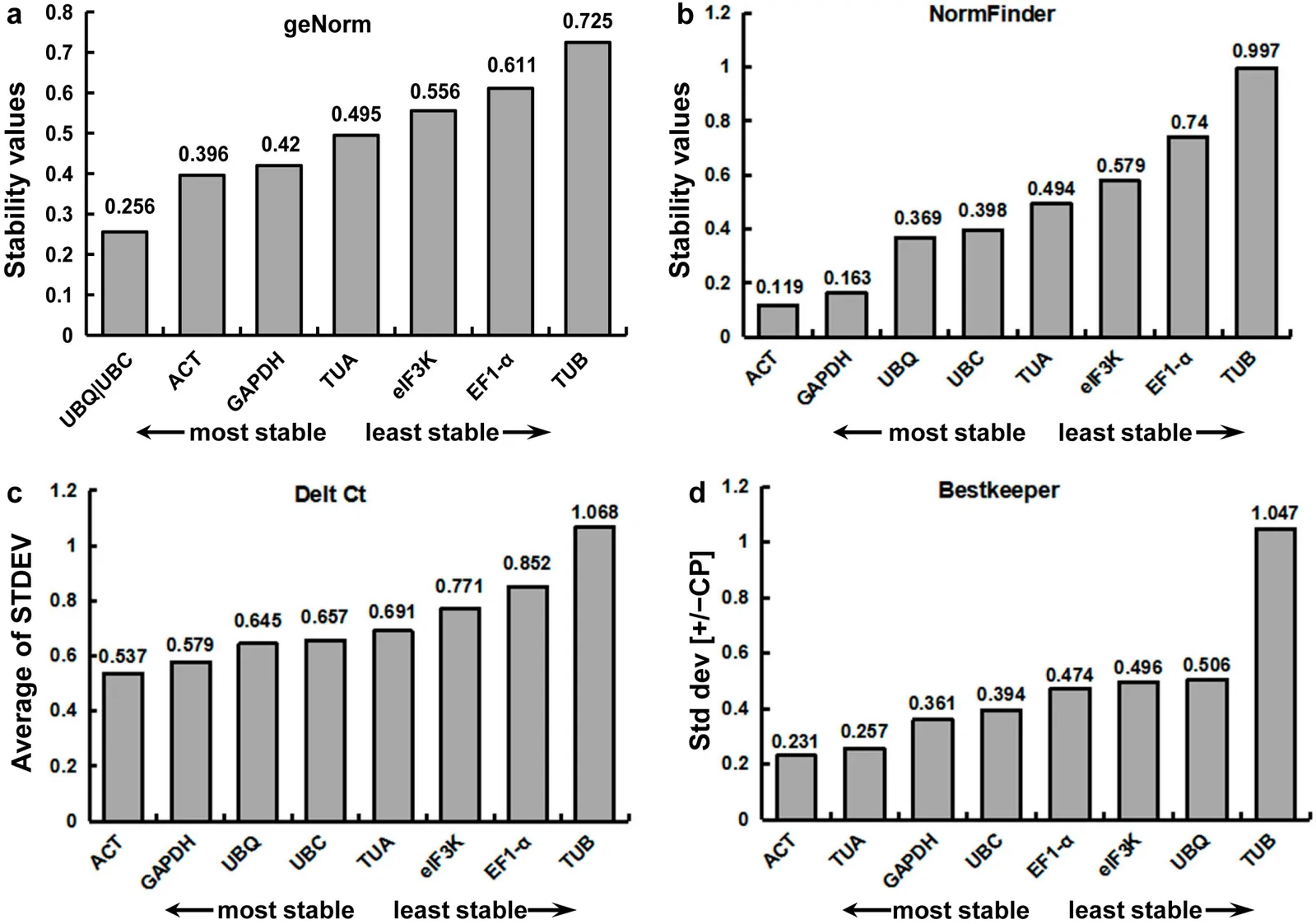
Expression stability analysis of eight candidate reference genes in Ws samples. Rank order was determined by geNorm (**a**), NormFinder (**b**), Comparative ΔCt (**c**) and BestKeeper (**d**). Ws, waterlogging stress.

The geNorm algorithm calculates the standard deviation (SD) of variation between pairs of reference genes after logarithmic transformation of the stability values (M value). A lower M value indicates higher gene expression stability [38]. Based on the geNorm analysis, all of the M values were below the cut-off value of 1.5 across all samples, demonstrating that all candidates exhibited acceptable expression stability (Figure 3a and Figure 4a). For Dt samples, the M values ranked in descending order were as follows: *eIF3K > TUB > UBQ > ACT > TUA > GAPDH > UBC/EF1-α*. *UBC* and *EF1-α* were the most stable reference genes, with the lowest M value of 0.272. For Ws samples, the M values ranked in descending order were as follows: *TUB > EF1-α > eIF3K > TUA > GAPDH > ACT > UBQ/UBC*. *UBQ* and *UBC* were the most stable reference genes, with the lowest M value of 0.256. *eIF3K* and *TUB* exhibited the least stability in Dt and Ws samples, with the highest M values of 0.792 and 0.725, respectively. Accordingly, *UBC* and *EF1-α* were the most suitable internal reference genes for different tissues of *E. stauntonii*, while *UBQ* and *UBC* were recommended for leaves under waterlogging stress.

**Figure 5:**
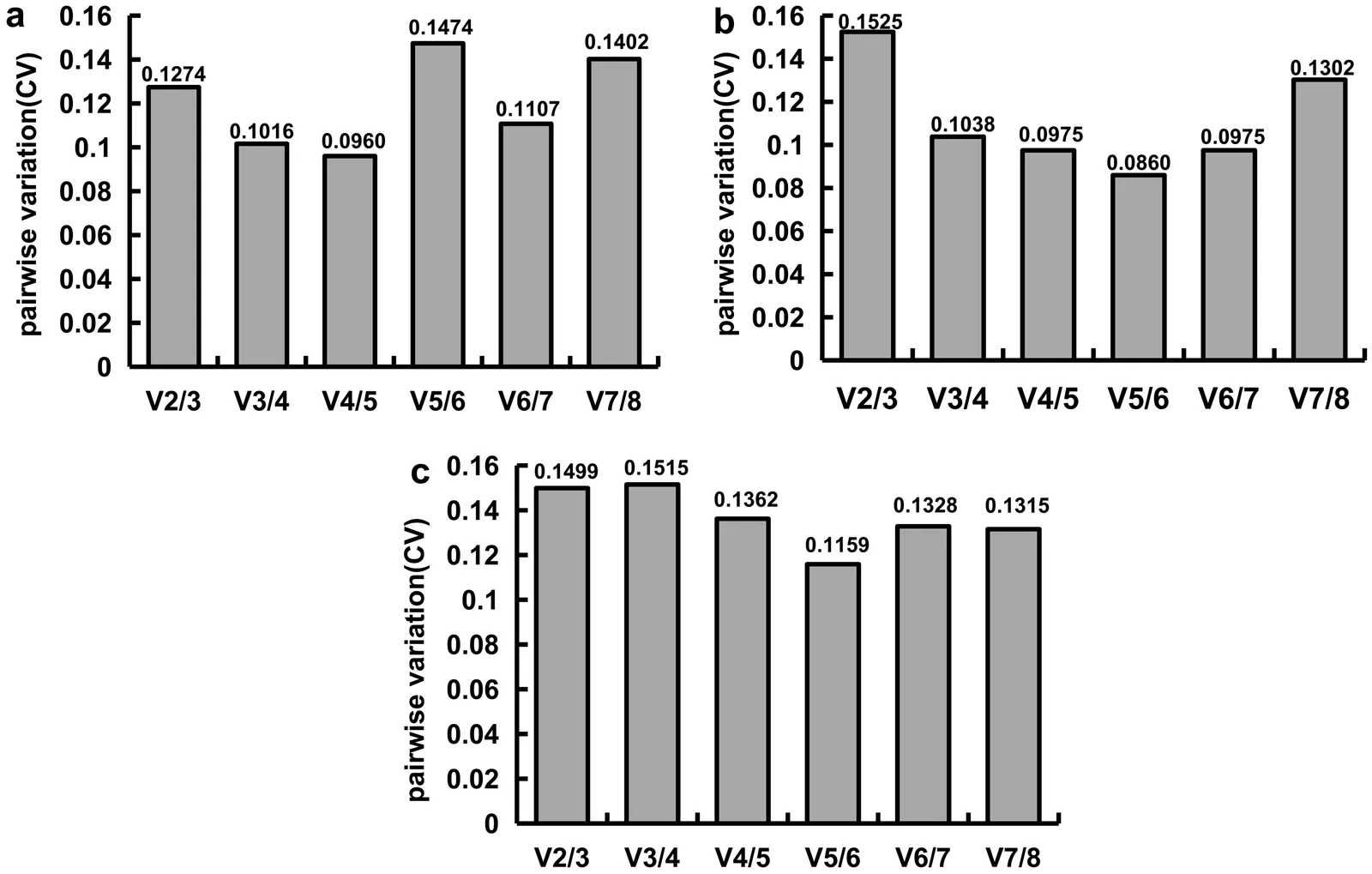
Determination of the optimal number of candidate reference genes in Dt samples (**a**), Ws samples (**b**) and the total samples (**c**). Dt, different tissues; Ws, waterlogging stress.

The geNorm program can calculate pairwise variation (Vn/Vn + 1) between sequentially ranked genes to determine the optimal number of reference genes required for accurate data normalization. The commonly adopted cut-off threshold is 0.15. For Dt samples, two reference genes were sufficient for qRT-PCR data normalization, as the V2/3 value (0.1008) was less than 0.15 (Figure 5a). For Ws samples, the V3/4 value (0.1038) was below 0.15, indicating that three reference genes should be employed in qRT-PCR data normalization (Figure 5b). For the total samples, two reference genes were recommended because the V2/3 value (0.1499) was less than 0.15 (Figure 5c).

#### 3.4.2. NormFinder Analysis

The NormFinder results are presented in Figure 3b and Figure 4b. Consistent with geNorm, a lower stability value indicates more stable gene expression. For Dt samples, the two most stable reference genes were *GAPDH* and *UBC*, with the lowest values of 0.103 and 0.222, respectively. This stability ranking differed slightly from the geNorm results. Nevertheless, *eIF3K*, with the highest value of 1.118, remained the least stable reference gene, ranking last among the candidate reference genes (Figure 3b). For Ws samples, *ACT*, *GAPDH*, and *UBQ* exhibited the best expression stability, with the lowest values of 0.119, 0.163, and 0.369, respectively. In comparison, *TUB* exhibited the lowest expression stability, with the highest value of 0.997 (Figure 4b). The different stability rankings of reference genes between Dt and Ws samples demonstrate that no single reference gene maintains absolutely stable expression across all experimental conditions in plants.

#### 3.4.3. Comparative ΔCt Analysis

Comparative ΔCt identifies stable reference genes based on pairwise comparisons of each pair of candidate genes within each sample [40]. Comparative ΔCt analysis yielded a stability pattern largely consistent with that obtained using the geNorm method. For Dt samples, *UBC* and *GAPDH* were also determined as the most stable reference genes, while *eIF3K* ranked as the least stable, with the highest standard deviation (STDEV) value (Figure 3c). For Ws samples, *ACT*, *GAPDH*, and *UBQ* were determined to be the most stable reference genes, and *TUB* was the least stable reference gene (Figure 4c).

#### 3.4.4. BestKeeper Analysis

BestKeeper evaluates gene expression stability using SD and the coefficient of variance (CV). Reference genes with an SD > 1 are regarded as unstable and were excluded from subsequent analysis [41]. Based on BestKeeper, *EF1-α* and *UBC* were identified as the most stable reference genes for Dt samples, with SD values of 0.673 and 0.738, respectively. However, *TUA*, *eIF3K*, *UBQ*, and *TUB*, with SD > 1, were classified as unstable genes (Figure 3d). For Ws samples, *TUB*, with SD > 1, was classified as an unstable gene. Other reference genes exhibited stable expression (SD < 1), among which *ACT*, *TUA*, and *GAPDH* performed best (Figure 4d). Under this algorithm, *TUB* remained the least stable reference gene in both the Dt and Ws groups.

#### 3.4.5. RefFinder Analysis

Owing to the inconsistency of gene expression stability ranking obtained from geNorm, NormFinder, Comparative ΔCt, and BestKeeper, it is necessary to integrate and evaluate these analytical results comprehensively. The web-based RefFinder analysis tool assigns an appropriate weight to each candidate gene and calculates the geometric mean of their weights to generate an overall stability ranking of the reference genes. As illustrated in Table 2, the overall stability ranking was *UBC > EF1-α > GAPDH > TUA > ACT > UBQ > TUB > eIF3K* for Dt samples and *ACT > GAPDH > UBQ > UBC > TUA > eIF3K > EF1-α > TUB* for Ws samples. For the total samples, the stability ranking was *GAPDH > ACT > UBC > TUA > EF1-α > eIF3K > UBQ > TUB*. Combined with the Vn/Vn+1 results from geNorm, *UBC* and *EF1-α* were the optimal reference genes for Dt samples, whereas *ACT*, *GAPDH*, and *UBQ* were recommended for Ws samples. For the total samples, *GAPDH* and *ACT* were identified as the two most suitable reference genes, while *TUB* was the least stable reference gene.

### 3.5. Validation of Reference Genes

**Figure 6:**
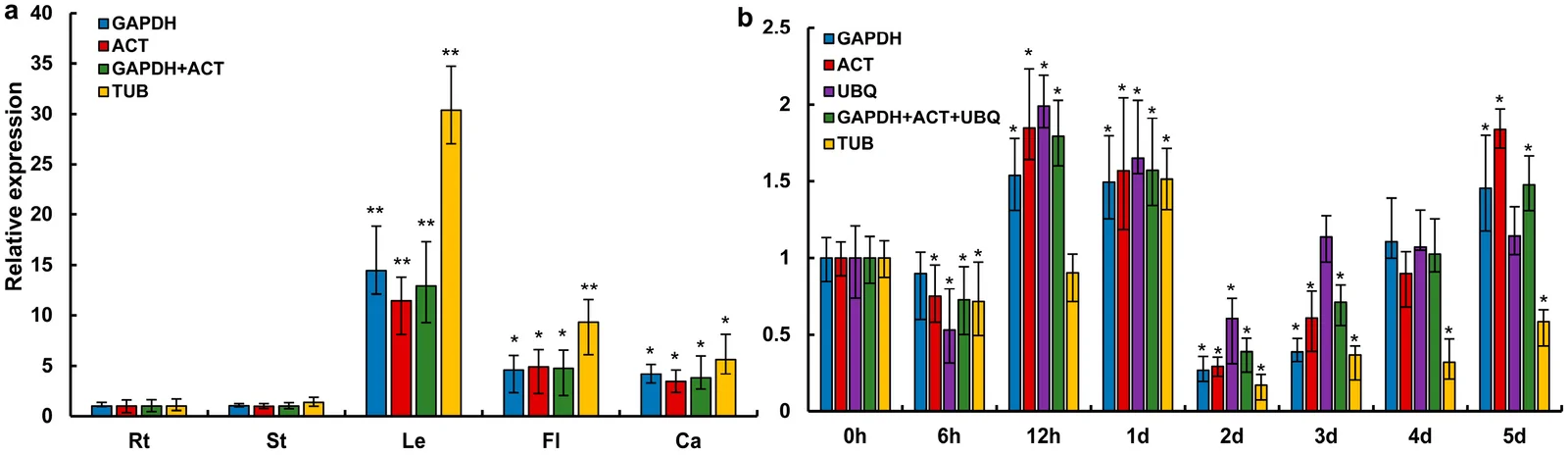
Relative expression patterns of *ElstPAL1* normalized using different reference genes. (**a**) relative expression levels of *ElstPAL1* in Dt samples; (**b**) relative expression levels of *ElstPAL1* in Ws samples. Dt, different tissues; Ws, waterlogging stress. Rt, root; St: stem; Le: leaf; Fl: Flower; Ca: Calli. Blue columns represent relative expression patterns of *ElstPAL1* normalized using *GAPDH*; red columns represent relative expression patterns of *ElstPAL1* normalized using *ACT*; purple columns represent relative expression patterns of *ElstPAL1* normalized using *UBQ*; yellow columns represent relative expression patterns of *ElstPAL1* normalized using *TUB*; green columns represent relative expression patterns of *ElstPAL1* normalized using *GAPDH* and *ACT* or *GAPDH*, *ACT* and *UBQ* in combination. Differences were considered statistically significant at *p* < 0.05 (*) and highly significant at *p* < 0.01 (**).

To validate the reliability of the selected reference genes, the expression patterns of *ElstPAL1* were examined in Dt samples and Ws samples. *TUB*, the least stable reference gene, was used as a negative control. As illustrated in Figure 6, the overall expression patterns of *ElstPAL1* exhibited identical trends in Dt samples, regardless of whether they were normalized individually using *GAPDH*, *ACT*, and *TUB* or in combination using *GAPDH + ACT*. The transcripts of *ElstPAL1* accumulated at a much higher level in leaves than in other tissues. The only difference among these results was the fold change, and the accumulation of *ElstPAL1* transcripts was markedly overestimated when normalized using *TUB*, the least stable reference gene (Figure 6a). For Ws samples, the same expression profiles of *ElstPAL1* were obtained whether normalized individually using *GAPDH*, *ACT*, *UBQ* or in combination, with the highest expression level observed at 12 h post-treatment. However, when *TUB* was used as the reference gene, the accumulation peak of *ElstPAL1* transcripts was detected at 1 d post-treatment, resulting in an expression pattern inconsistent with those observed using stable reference genes (Figure 6b). These results indicated that gene expression patterns were significantly influenced when unstable reference genes were used for qRT-PCR data normalization. Moreover, *UBC* and *EF1-α* were the best reference genes in different tissues; however, the expression patterns of *ElstPAL1* normalized with these genes were highly consistent with those obtained using *GAPDH* and *ACT* (Figure S2). Considering the experimental cost and operational efficiency, the combination of *GAPDH* and *ACT* was recommended to normalize qRT-PCR data in *E. stauntonii* for different tissues and total samples, while the combination of *GAPDH*, *ACT*, and *UBQ* was suitable for leaves under waterlogging stress.

## 4. Discussion

Gene expression analysis provides crucial insights into gene function in plant growth, development, and responses to external stimuli. Among current analytical approaches, qRT-PCR is widely recognized as a powerful and efficient technique for gene expression quantification in various plant species [4,44]. Reliable qRT-PCR quantification heavily depends on accurate data normalization using highly stable reference genes, thereby ensuring the credibility of experimental results [45]. Accordingly, an essential first step is to identify suitable reference genes. Although reference genes have been established in many plants, little information is available regarding reference genes of *E. stauntonii*. In this study, eight reference genes were selected, and their expression stability was evaluated in different tissues and in leaves under waterlogging stress. These findings will provide a foundation for subsequent molecular functional studies in *E. stauntonii*.

Four commonly used statistical algorithms, namely, geNorm, NormFinder, Comparative ΔCt, and BestKeeper, were employed to validate the expression stability of candidate reference genes [46]. According to the geNorm algorithm, the two most stable reference genes in different tissues were *UBC* and *EF1-α*, whereas *UBQ* and *UBC* were the most stable reference genes in leaves under waterlogging stress. NormFinder identified *GAPDH* and *UBC* as the most stable for different tissues, and *ACT* and *GAPDH* for waterlogging stress. The Comparative ΔCt method ranked *UBC* and *GAPDH* as the most stable for different tissues, and *ACT* and *GAPDH* for waterlogging stress. BestKeeper, in turn, recommended *EF1-α* and *UBC* for different tissues, and *ACT* and *TUA* for waterlogging stress. It is evident that the optimal reference genes identified using the four algorithms exhibited both consistencies and discrepancies. Similar results have been documented in multiple plant species, including *P. ginseng* [4], *Sagittaria trifolia* [47], *Euonymus japonicus* [35], and *Broussonetia papyrifera* [48]. Such discrepancies can be attributed to their different underlying principles. Each tool adopts a unique computational framework to assess expression variation. They focus on different dimensions of gene expression stability, which inevitably leads to divergent rankings. Thus, to avoid the bias of any single analytical approach, a comprehensive evaluation should be performed. RefFinder is capable of calculating the geometric mean of the stability value weights derived from multiple algorithms and generating a comprehensive stability ranking [46]. Owing to its advantage of comprehensive analysis, RefFinder has been widely used for screening and evaluating reference genes in various plant species [11,49,50]. RefFinder analysis revealed distinct optimal reference genes for different experimental conditions. Specifically, *UBC*, *ACT*, and *GAPDH* were identified as the top-ranked reference genes for different tissues, waterlogging-stressed leaves and the total samples, respectively. In addition, *EF1-α*, *GAPDH*, and *ACT* also exhibited favorable expression stability and served as the second credible reference genes for the three experimental sample sets. Further validation based on the expression profiles of *ElstPAL1* demonstrated their reliability for qRT-PCR data normalization. In addition, the combined use of *UBC* + *EF1-α* or *GAPDH* + *ACT* exhibited negligible effects on qRT-PCR data normalization in different tissues. Considering experimental cost and operational efficiency, the combination of *GAPDH* and *ACT* was ultimately recommended for different tissues and total samples in this study.

Glyceraldehyde-3-phosphate dehydrogenase (GAPDH), a key enzyme involved in the glycolysis pathway, has been demonstrated to participate in many biological processes, such as gene expression regulation, cellular signal transduction, and environmental stress adaptation [51]. Actin (ACT) is an indispensable component of the cytoskeleton, playing a pivotal role in almost all plant growth and developmental processes [52,53]. *GAPDH* and *ACT* have emerged as optimal reference genes in various plants, such as in different *Arabidopsis pumila* tissues and leaves under salt stress [54], diverse *Schima superba* tissues [55], and *Eremochloa ophiuroides* stems under multiple stresses of phosphorus deficiency and/or aluminum toxicity [56], in addition to Chinese fir roots under drought stress and phosphorus starvation [46]. In our study, they were also the most stable reference genes in *E. stauntonii*. *UBQ*, a common housekeeping gene, encodes ubiquitin, which is mainly involved in intracellular protein degradation. It has been widely used as a reference gene in various plants, such as *Amorphophallus Konjac* [57], *Miscanthus lutarioriparia* [58], and *Eucalyptus* [59]. Inclusion of *UBQ* can further improve the accuracy of qRT-PCR normalization for waterlogging-stressed leaves, and the combination of *GAPDH*, *ACT*, and *UBQ* was suitable for this stress condition. However, β-tubulin (*TUB*) proved to be the least stable reference gene across all tested samples from *E. stauntonii*. Similar results were observed in *P. tomentosa* stems at different developmental phases [37], in female and male flowers of *Salix suchowensis* [60], and in different tissues of *Eichhornia crassipes* [61]. As a major constituent of microtubules, tubulin participates in cytoskeleton remodeling [62]. Under waterlogging stress, plants undergo active cytoskeleton rearrangement to adapt to hypoxic environments and adjust cell morphology, which leads to significant transcriptional fluctuation of *TUB*. This biological characteristic may account for its poor performance as an internal control in *E. stauntonii* waterlogging treatment.

It should be emphasized that these recommendations are confined to the specific tissues and waterlogging conditions investigated in this study, and their applicability under other abiotic stresses, different developmental stages, or biotic challenges remains to be determined. Moreover, the validation of the selected reference genes was performed using only a single target gene (*ElstPAL1*). Further validation with a broader panel of target genes with diverse expression profiles would strengthen the general applicability of these findings. It is also worth noting that transcriptome data enable high-throughput screening of candidate internal reference genes with low expression variability; however, this approach is not without inherent limitations. For instance, RNA-seq data are affected by sequencing depth, alignment efficiency, and transcript variable splicing, which can result in incomplete identification of candidate reference genes and potentially exclude certain promising low-expression reference genes. Consequently, transcriptome data can only serve as an initial screening pool, and the suitable reference genes must undergo rigorous validation and evaluation through subsequent qRT-PCR experiments. Collectively, our findings highlight that selecting the appropriate reference genes remains a paramount prerequisite for normalizing target gene expression via qRT-PCR in specific organisms under different conditions.

## 5. Conclusions

In summary, eight candidate reference genes were selected in *E. stauntonii* and their expression stability was systematically evaluated in two distinct groups. The qRT-PCR data were comprehensively analyzed using five independent algorithms: geNorm, NormFinder, Comparative ΔCt, BestKeeper, and RefFinder. Ultimately, *GAPDH* and *ACT* were recommended as the optimal reference genes for different tissues and total samples, whereas *GAPDH*, *ACT* and *UBQ* were suitable for leaves under waterlogging stress. To the best of our knowledge, this study is the first systematic evaluation and validation of internal reference genes for qRT-PCR data normalization in *E. stauntonii.* These results provide a valuable reference for future gene expression studies and lay a foundation for subsequent investigations into the molecular mechanisms underlying stress responses in this economically important species.

## Figures and Tables

**Table 1:** The primer information and PCR amplification of eight reference genes and *PAL1* in *E. stauntonii*.

Gene	Gene Description	Primer Sequence (5′-3′) (Forward/Reverse)	Product Tm (°C)	Product (bp)	E(%)	R^2^
Reference genes						
*GAPDH*	glyceraldehyde-3-phosphate dehydrogenase	F: TCCGTGACTGTCTTTGGCTT	81	110	99.8	0.9939
		R: GCGGCCTTATCCTTGTCAGT				
*ACT*	actin	F: CCTCTCAACCCCAAGGCAAA	81.5	150	107.8	0.9959
		R: ACCATCACCAGAATCGAGCA				
*UBQ*	ubiquitin	F: GGGCGTGGATACATAGGAAGG	81	107	105.2	0.994
		R: TTCACCCGAACCTTCTTCCC				
*UBC*	ubiquitin-conjugating enzyme	F: GAACAGCAATTTACCGCGCA	82	102	103.6	0.9937
		R: TACCGCATGTTGTCTTCCGA				
*TUA*	α-tubulin	F: TCCCTGCTTCTTGAGCGTTT	81	130	100.5	0.9996
		R: GGGAGTGAGTGGACAAGACG				
*TUB*	β-tubulin	F: GGGCACCCTTCTCATTTCCA	81.5	130	94.4	0.9907
		R: GTGCACAGACAGAGTAGCGT				
*EF1-α*	elongation factor 1α	F: ATGCACCACGAGTCTATGGC	81.5	100	102.8	0.9934
		R: AGGCAACAAAACCACGCTTC				
*eIF3K*	Eukaryotic translation initiation factor 3 subunit K	F: GTCGCCGTCAATCCCTACAA	81.5	109	103.4	0.9932
		R: GCAGAAGGCAAAGATTCGCA				
Target gene						
*PAL1*	phenylalanine ammonia-lyase 1	F: TCACATAGGAGGACCAGCCA	81.5	113	106.8	0.999
		R: GCCGAGTGAGGTAGAGTGTG				

**Table 2:** Comprehensive stability ranking of the eight candidate reference genes.

Method	1	2	3	4	5	6	7	8
Dt samples	Ranking Order (Excellent–Good–Average)							
geNorm	*UBC/EF1-α*		*GAPDH*	*TUA*	*ACT*	*UBQ*	*TUB*	*eIF3K*
NormFinder	*GAPDH*	*UBC*	*TUA*	*EF1-α*	*ACT*	*UBQ*	*TUB*	*eIF3K*
Delta Ct	*UBC*	*GAPDH*	*TUA*	*EF1-α*	*ACT*	*UBQ*	*TUB*	*eIF3K*
BestKeeper	*EF1-α*	*UBC*	*ACT*	*GAPDH*	*TUA*	*eIF3K*	*UBQ*	*TUB*
RefFinder	*UBC*	*EF1-α*	*GAPDH*	*TUA*	*ACT*	*UBQ*	*TUB*	*eIF3K*
Ws samples								
geNorm	*UBQ/UBC*		*ACT*	*GAPDH*	*TUA*	*eIF3K*	*EF1-α*	*TUB*
NormFinder	*ACT*	*GAPDH*	*UBQ*	*UBC*	*TUA*	*eIF3K*	*EF1-α*	*TUB*
Delta Ct	*ACT*	*GAPDH*	*UBQ*	*UBC*	*TUA*	*eIF3K*	*EF1-α*	*TUB*
BestKeeper	*ACT*	*TUA*	*GAPDH*	*UBC*	*EF1-α*	*eIF3K*	*UBQ*	*TUB*
RefFinder	*ACT*	*GAPDH*	*UBQ*	*UBC*	*TUA*	*eIF3K*	*EF1-α*	*TUB*
Total samples								
geNorm	*GAPDH/ACT*		*EF1-α*	*UBC*	*TUA*	*eIF3K*	*UBQ*	*TUB*
NormFinder	*GAPDH*	*ACT*	*UBC*	*TUA*	*EF1-α*	*eIF3K*	*UBQ*	*TUB*
Delta Ct	*GAPDH*	*ACT*	*UBC*	*TUA*	*EF1-α*	*eIF3K*	*UBQ*	*TUB*
BestKeeper	*UBC*	*TUA*	*GAPDH*	*ACT*	*EF1-α*	*eIF3K*	*UBQ*	*TUB*
RefFinder	*GAPDH*	*ACT*	*UBC*	*TUA*	*EF1-α*	*eIF3K*	*UBQ*	*TUB*

## Data Availability

The original contributions presented in this study are included in the article/Supplementary Materials. Further inquiries can be directed to the corresponding author.

## Supplementary Materials

The following supporting information can be downloaded at: https://www.mdpi.com/article/10.3390/cimb48090933/s1.
