# Supplementary material for: Selection and Validation of qRT-PCR Reference Genes in Elsholtzia stauntonii
Source: Curr Issues Mol Biol. 2026 Sep 12;48(9):933. doi: 10.3390/cimb48090933 (PMC13605697; doi:10.3390/cimb48090933)
Supplement: Supplementary file 1 [file cimb-48-00933-s001.zip › cimb-4448189-supplementary.pdf]

Supplementary Materials

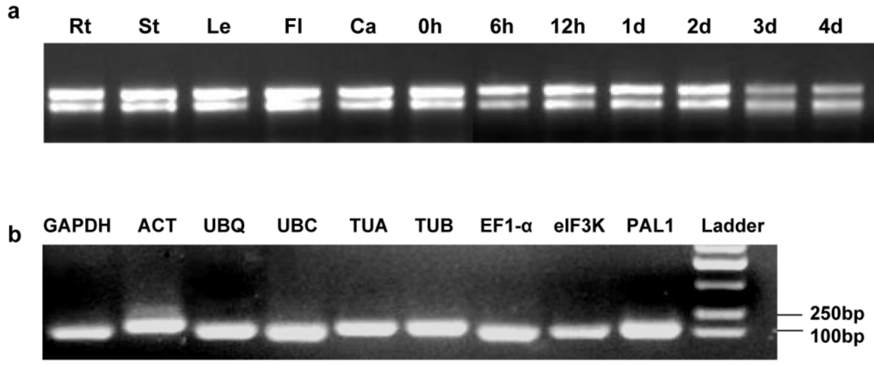

Figure S1. The assessment of RNA quality and primer specificity by agarose-gel electrophoresis. a. Agarose gel electrophoresis of RNA; b. Agarose gel electrophoresis of PCR products of 8 candidate reference genes and target gene PAL1

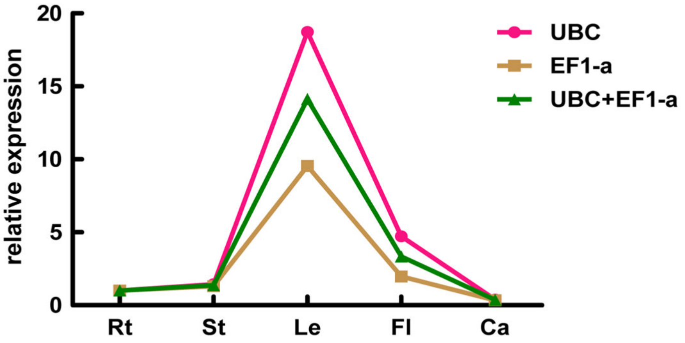

Figure S2. Relative expression patterns of *ElstPAL1* normalized using *UBC* and *EF1-α*. Rt, root; St: stem; Le: leaf; Fl: Flower; Ca: Calli; Pink curve represents relative expression patterns of *ElstPAL1* normalized using *UBC*; Brown curve represents relative expression patterns of *ElstPAL1* normalized using *EF1-α*; Green curve represents relative expression patterns of *ElstPAL1* normalized using *UBC* and *EF1-α* in combination.
